# *N*-(2,3-Dimethyl­phen­yl)-4-fluoro-*N*-[(4-fluoro­phen­yl)sulfon­yl]benzene­sulfonamide

**DOI:** 10.1107/S1600536812039402

**Published:** 2012-09-22

**Authors:** Shumaila Younas Mughal, Islam Ullah Khan, William T. A. Harrison, Muneeb Hayat Khan, Muhammad Nawaz Tahir

**Affiliations:** a Materials Chemistry Laboratry, Department of Chemistry, GC University, Lahore 54000, Pakistan; b Department of Chemistry, University of Aberdeen, Meston Walk, Aberdeen AB24 3UE, Scotland; c Questioned Documents Unit, Punjab Forensic Science Agency, Home Department, Lahore, Pakistan; d Department of Physics, University of Sargodha, Punjab, Pakistan

## Abstract

In the title compound, C_20_H_17_F_2_NO_4_S_2_, the dihedral angles between the *o*-xylene ring and the fluoro­benzene rings are 31.7 (1) and 32.8 (1)°, and the dihedral angle between the fluoro­benzene rings is 50.9 (1)°. The C—N—S—C torsion angles are 76.7 (2) and 101.8 (2)°. In the crystal, mol­ecules are connected by C—H⋯O inter­actions into sheets in the *ab* plane.

## Related literature

For related crystal structures, see: Hanson & Hitchcock (2004 ▶); Low *et al.* (2006 ▶); Mughal *et al.* (2012 ▶).
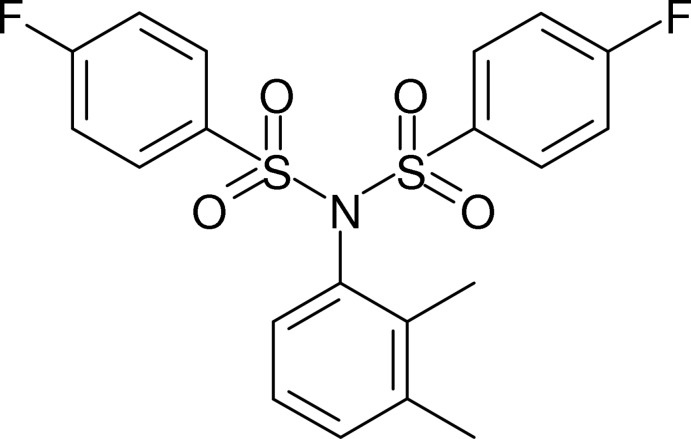


## Experimental

### 

#### Crystal data

C_20_H_17_F_2_NO_4_S_2_
*M* *_r_* = 437.47
Orthorhombic, 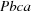

*a* = 9.9493 (5) Å
*b* = 14.7107 (8) Å
*c* = 26.6871 (17) Å
*V* = 3906.0 (4) Å^3^
*Z* = 8
Mo *K*α radiation
μ = 0.32 mm^−1^
*T* = 296 K
0.28 × 0.25 × 0.23 mm

#### Data collection

Bruker APEXII CCD diffractometer
Absorption correction: multi-scan (*SADABS*; Sheldrick, 2008 ▶) *T*_min_ = 0.916, *T*_max_ = 0.930
31734 measured reflections
4352 independent reflections
2943 reflections with *I* > 2σ(*I*)
*R*_int_ = 0.046

#### Refinement

*R*[*F* ^2^ > 2σ(*F* ^2^)] = 0.048
*wR*(*F* ^2^) = 0.133
*S* = 1.02
4352 reflections
264 parameters
H-atom parameters constrained
Δρ_max_ = 0.47 e Å^−3^
Δρ_min_ = −0.29 e Å^−3^

### 

Data collection: *APEX2* (Bruker, 2007 ▶); cell refinement: *SAINT* (Bruker, 2007 ▶); data reduction: *SAINT*; program(s) used to solve structure: *SHELXS97* (Sheldrick, 2008 ▶); program(s) used to refine structure: *SHELXL97* (Sheldrick, 2008 ▶); molecular graphics: *ORTEP-3* (Farrugia, 1997 ▶); software used to prepare material for publication: *SHELXL97*.

## Supplementary Material

Crystal structure: contains datablock(s) global, I. DOI: 10.1107/S1600536812039402/ld2072sup1.cif

Structure factors: contains datablock(s) I. DOI: 10.1107/S1600536812039402/ld2072Isup2.hkl

Supplementary material file. DOI: 10.1107/S1600536812039402/ld2072Isup3.cml

Additional supplementary materials:  crystallographic information; 3D view; checkCIF report

## Figures and Tables

**Table 1:** Hydrogen-bond geometry (Å, °)

*D*—H⋯*A*	*D*—H	H⋯*A*	*D*⋯*A*	*D*—H⋯*A*
C8—H8*A*⋯O2^i^	0.96	2.57	3.517 (5)	170
C19—H19⋯O1^ii^	0.93	2.39	3.260 (4)	157

Symmetry codes: (i) 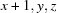; (ii) 
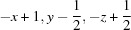
.

**Fig. 1.**
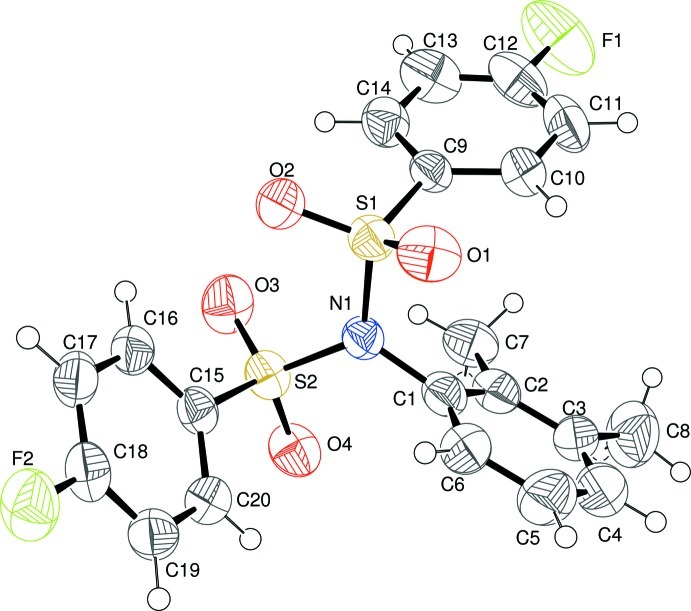
The molecular structure of (I), showing displacement ellipsoids at the 50% probability level.

## supplementary crystallographic information

### Comment

Rather than the intended 4-fluoro-*N*-(2,3-dimethylphenyl)benzenesulfonamide, the title `double' sulfonamide (I), (Fig. 1) was unexpectedly obtained in our ongoing studies (Mughal *et al.*, 2012). The related structures of 4-chloroaniline-*N*,*N*-ditoluene-*p*-sulfonamide, (II) (Hanson & Hitchcock, 2004) and 4-iodo-*N*,*N*-bis-(2-nitrophenylsulfonyl)aniline, (III) (Low *et al.*, 2006) have been described previously.

The molecule of (I) cannot possess any local symmetry due to the two methyl groups of the xylene ring. Without them, the rest of the moleucle possesses approximate local twofold symmetry about the C1—N1 axis.

The sulfonamide C—N—S—C torsion angles in (I) [76.7 (2) and 101.8 (2)°] compare well to the corresponding value of 80.2° reported in (II), where the molecule posesses a crystallographic twofold symmetry. The respective values in (III) are 98.9 (2) and 94.3 (2)°. The bond-angle sum for the N atom in (I) of 359.7° implies *sp*^2^ hybridization for the N atom.

In the crystal of (I), there are weak C—H···O hydrogen bonds between the molecules (Table 1). The C8 bond results in translational chains along [100] and the C19 bond results in helices along [010]. Together these generate (001) sheets.

### Experimental

0.10 g of 2,3-dimethyl aniline was dissolved in 15 ml dichloromethane, and 0.16 g of 4-fluoro benzene sulfonyl chloride was added. The mixture was stirred at room temperature overnight and its pH was maintained at 8–9 with triethylamine. On completion of the reaction (after TLC), 1*M* HCl solution was added, the organic fraction was separated, and the solvent was evaporated at room temperature to generate dark brown crystals in 94% yield. Yellow blocks were recrystallized from ethanol solution.

### Refinement

The H atoms were placed in calculated positions (C—H = 0.93–0.96 Å) and refined as riding with *U*_iso_(H) = 1.2*U*_eq_(C) or 1.5*U*_eq_(methyl C). The methyl groups were allowed to rotate to fit the electron density.

### Crystal data

**Table:**

C_20_H_17_F_2_NO_4_S_2_	*D*_x_ = 1.488 Mg m^−^^3^
*M**_r_* = 437.47	Mo *K*α radiation, λ = 0.71073 Å
Orthorhombic, *P**b**c**a*	Cell parameters from 202 reflections
*a* = 9.9493 (5) Å	θ = 3.2–20.5°
*b* = 14.7107 (8) Å	µ = 0.32 mm^−^^1^
*c* = 26.6871 (17) Å	*T* = 296 K
*V* = 3906.0 (4) Å^3^	Block, yellow
*Z* = 8	0.28 × 0.25 × 0.23 mm
*F*(000) = 1808	

### Data collection

**Table:**

Bruker APEXII CCD diffractometer	4352 independent reflections
Radiation source: fine-focus sealed tube	2943 reflections with *I* > 2σ(*I*)
Graphite monochromator	*R*_int_ = 0.046
ω scans	θ_max_ = 27.3°, θ_min_ = 1.5°
Absorption correction: multi-scan (*SADABS*; Sheldrick, 2008)	*h* = −12→12
*T*_min_ = 0.916, *T*_max_ = 0.930	*k* = −18→14
31734 measured reflections	*l* = −34→34

### Refinement

**Table:**

Refinement on *F*^2^	Primary atom site location: structure-invariant direct methods
Least-squares matrix: full	Secondary atom site location: difference Fourier map
*R*[*F*^2^ > 2σ(*F*^2^)] = 0.048	Hydrogen site location: inferred from neighbouring sites
*wR*(*F*^2^) = 0.133	H-atom parameters constrained
*S* = 1.02	*w* = 1/[σ^2^(*F*_o_^2^) + (0.0524*P*)^2^ + 2.7378*P*] where *P* = (*F*_o_^2^ + 2*F*_c_^2^)/3
4352 reflections	(Δ/σ)_max_ < 0.001
264 parameters	Δρ_max_ = 0.47 e Å^−^^3^
0 restraints	Δρ_min_ = −0.29 e Å^−^^3^

### Special details

**Table:**

Geometry. All e.s.d.'s (except the e.s.d. in the dihedral angle between two l.s. planes) are estimated using the full covariance matrix. The cell e.s.d.'s are taken into account individually in the estimation of e.s.d.'s in distances, angles and torsion angles; correlations between e.s.d.'s in cell parameters are only used when they are defined by crystal symmetry. An approximate (isotropic) treatment of cell e.s.d.'s is used for estimating e.s.d.'s involving l.s. planes.
Refinement. Refinement of *F*^2^ against ALL reflections. The weighted *R*-factor *wR* and goodness of fit *S* are based on *F*^2^, conventional *R*-factors *R* are based on *F*, with *F* set to zero for negative *F*^2^. The threshold expression of *F*^2^ > σ(*F*^2^) is used only for calculating *R*-factors(gt) *etc*. and is not relevant to the choice of reflections for refinement. *R*-factors based on *F*^2^ are statistically about twice as large as those based on *F*, and *R*-factors based on ALL data will be even larger.

### Fractional atomic coordinates and isotropic or equivalent isotropic displacement parameters (Å^2^)

**Table:**

	*x*	*y*	*z*	*U*_iso_*/*U*_eq_	
C1	0.6678 (3)	0.45285 (18)	0.13773 (11)	0.0504 (7)	
C2	0.7555 (3)	0.48512 (18)	0.10252 (10)	0.0540 (7)	
C3	0.8910 (3)	0.4952 (2)	0.11660 (13)	0.0616 (8)	
C4	0.9311 (4)	0.4696 (2)	0.16383 (14)	0.0717 (9)	
H4	1.0208	0.4763	0.1729	0.086*	
C5	0.8414 (4)	0.4343 (2)	0.19804 (14)	0.0765 (10)	
H5	0.8715	0.4167	0.2296	0.092*	
C6	0.7092 (3)	0.4250 (2)	0.18605 (11)	0.0595 (8)	
H6	0.6480	0.4011	0.2089	0.071*	
C7	0.7112 (3)	0.5083 (2)	0.05128 (11)	0.0666 (9)	
H7A	0.7561	0.4696	0.0276	0.100*	
H7B	0.7328	0.5706	0.0443	0.100*	
H7C	0.6158	0.4996	0.0486	0.100*	
C8	0.9920 (4)	0.5330 (3)	0.08077 (16)	0.0961 (13)	
H8A	1.0796	0.5312	0.0958	0.144*	
H8B	0.9691	0.5947	0.0729	0.144*	
H8C	0.9922	0.4973	0.0506	0.144*	
C9	0.4640 (3)	0.62628 (17)	0.11107 (10)	0.0451 (6)	
C10	0.5733 (3)	0.68129 (19)	0.12047 (12)	0.0571 (7)	
H10	0.6275	0.6707	0.1483	0.069*	
C11	0.6018 (4)	0.7518 (2)	0.08853 (14)	0.0733 (9)	
H11	0.6744	0.7902	0.0943	0.088*	
C12	0.5205 (4)	0.7638 (2)	0.04815 (14)	0.0768 (10)	
C13	0.4101 (4)	0.7119 (2)	0.03854 (12)	0.0749 (10)	
H13	0.3554	0.7241	0.0111	0.090*	
C14	0.3816 (3)	0.6414 (2)	0.07036 (10)	0.0583 (7)	
H14	0.3077	0.6041	0.0646	0.070*	
C15	0.3779 (3)	0.29685 (18)	0.14708 (9)	0.0449 (6)	
C16	0.2445 (3)	0.31051 (19)	0.15903 (10)	0.0517 (7)	
H16	0.1919	0.3501	0.1402	0.062*	
C17	0.1902 (3)	0.2645 (2)	0.19928 (11)	0.0557 (7)	
H17	0.1007	0.2728	0.2083	0.067*	
C18	0.2703 (3)	0.2067 (2)	0.22544 (10)	0.0542 (7)	
C19	0.4012 (3)	0.1897 (2)	0.21330 (11)	0.0569 (7)	
H19	0.4518	0.1480	0.2314	0.068*	
C20	0.4558 (3)	0.23611 (19)	0.17354 (10)	0.0518 (7)	
H20	0.5450	0.2265	0.1645	0.062*	
S1	0.43137 (7)	0.53555 (5)	0.15106 (3)	0.0483 (2)	
S2	0.45376 (7)	0.35921 (5)	0.09885 (2)	0.0494 (2)	
F1	0.5499 (3)	0.83173 (15)	0.01607 (9)	0.1272 (10)	
F2	0.21844 (19)	0.16374 (13)	0.26576 (7)	0.0773 (6)	
O1	0.4877 (2)	0.55627 (14)	0.19840 (7)	0.0654 (6)	
O2	0.29538 (19)	0.50962 (14)	0.14719 (9)	0.0677 (6)	
O3	0.3540 (2)	0.39068 (16)	0.06534 (7)	0.0704 (6)	
O4	0.5668 (2)	0.30948 (14)	0.08134 (7)	0.0622 (5)	
N1	0.5226 (2)	0.44974 (14)	0.12742 (8)	0.0444 (5)	

### Atomic displacement parameters (Å^2^)

**Table:**

	*U*^11^	*U*^22^	*U*^33^	*U*^12^	*U*^13^	*U*^23^
C1	0.0518 (16)	0.0389 (14)	0.0604 (17)	−0.0005 (12)	0.0075 (13)	−0.0049 (12)
C2	0.0611 (17)	0.0435 (16)	0.0574 (17)	0.0019 (13)	0.0051 (14)	−0.0018 (13)
C3	0.0486 (16)	0.0509 (17)	0.085 (2)	0.0025 (14)	−0.0022 (16)	−0.0086 (16)
C4	0.064 (2)	0.063 (2)	0.088 (2)	−0.0010 (17)	0.0044 (19)	−0.0038 (18)
C5	0.081 (2)	0.080 (2)	0.068 (2)	0.006 (2)	−0.0230 (19)	0.0015 (18)
C6	0.0580 (18)	0.0585 (18)	0.0619 (18)	−0.0002 (15)	−0.0060 (14)	0.0022 (14)
C7	0.083 (2)	0.0597 (19)	0.0573 (18)	0.0066 (17)	0.0038 (16)	0.0049 (15)
C8	0.071 (2)	0.105 (3)	0.112 (3)	−0.021 (2)	0.015 (2)	0.003 (3)
C9	0.0491 (15)	0.0359 (13)	0.0502 (14)	−0.0006 (12)	0.0011 (12)	−0.0053 (11)
C10	0.0583 (18)	0.0478 (16)	0.0653 (18)	−0.0045 (14)	−0.0030 (15)	−0.0058 (14)
C11	0.082 (2)	0.0494 (18)	0.089 (3)	−0.0206 (17)	0.008 (2)	−0.0050 (17)
C12	0.115 (3)	0.0473 (18)	0.068 (2)	−0.007 (2)	0.017 (2)	0.0057 (16)
C13	0.109 (3)	0.068 (2)	0.0487 (17)	0.008 (2)	−0.0091 (18)	0.0019 (16)
C14	0.0642 (18)	0.0571 (18)	0.0534 (17)	−0.0027 (15)	−0.0054 (14)	−0.0081 (14)
C15	0.0503 (15)	0.0404 (14)	0.0440 (14)	−0.0087 (12)	−0.0049 (12)	−0.0045 (11)
C16	0.0473 (15)	0.0484 (16)	0.0594 (17)	−0.0078 (13)	−0.0102 (13)	0.0035 (13)
C17	0.0451 (15)	0.0568 (17)	0.0651 (18)	−0.0104 (14)	0.0022 (14)	−0.0016 (14)
C18	0.0577 (18)	0.0554 (17)	0.0496 (15)	−0.0180 (14)	−0.0034 (13)	0.0017 (13)
C19	0.0578 (18)	0.0541 (17)	0.0588 (17)	−0.0048 (14)	−0.0106 (14)	0.0102 (14)
C20	0.0458 (15)	0.0510 (16)	0.0585 (17)	−0.0007 (13)	−0.0005 (13)	0.0005 (13)
S1	0.0486 (4)	0.0448 (4)	0.0516 (4)	0.0001 (3)	0.0086 (3)	−0.0025 (3)
S2	0.0592 (4)	0.0494 (4)	0.0397 (3)	−0.0057 (3)	−0.0004 (3)	−0.0028 (3)
F1	0.209 (3)	0.0732 (14)	0.0998 (17)	−0.0283 (17)	0.0147 (18)	0.0319 (13)
F2	0.0742 (12)	0.0903 (14)	0.0672 (11)	−0.0198 (10)	0.0045 (10)	0.0228 (10)
O1	0.0839 (15)	0.0678 (13)	0.0445 (11)	0.0104 (12)	0.0046 (10)	−0.0086 (9)
O2	0.0462 (11)	0.0566 (13)	0.1003 (17)	−0.0017 (10)	0.0155 (11)	0.0027 (11)
O3	0.0764 (15)	0.0838 (15)	0.0510 (11)	−0.0119 (12)	−0.0174 (11)	0.0096 (11)
O4	0.0746 (14)	0.0565 (12)	0.0556 (12)	−0.0007 (11)	0.0155 (10)	−0.0153 (10)
N1	0.0429 (11)	0.0399 (12)	0.0503 (12)	−0.0012 (9)	0.0031 (10)	−0.0043 (10)

### Geometric parameters (Å, º)

**Table:**

C1—C2	1.367 (4)	C11—H11	0.9300
C1—C6	1.414 (4)	C12—F1	1.348 (4)
C1—N1	1.471 (4)	C12—C13	1.362 (5)
C2—C3	1.407 (4)	C13—C14	1.371 (4)
C2—C7	1.476 (4)	C13—H13	0.9300
C3—C4	1.375 (5)	C14—H14	0.9300
C3—C8	1.495 (5)	C15—C20	1.377 (4)
C4—C5	1.378 (5)	C15—C16	1.380 (4)
C4—H4	0.9300	C15—S2	1.751 (3)
C5—C6	1.361 (4)	C16—C17	1.380 (4)
C5—H5	0.9300	C16—H16	0.9300
C6—H6	0.9300	C17—C18	1.358 (4)
C7—H7A	0.9600	C17—H17	0.9300
C7—H7B	0.9600	C18—F2	1.350 (3)
C7—H7C	0.9600	C18—C19	1.365 (4)
C8—H8A	0.9600	C19—C20	1.374 (4)
C8—H8B	0.9600	C19—H19	0.9300
C8—H8C	0.9600	C20—H20	0.9300
C9—C10	1.378 (4)	S1—O2	1.410 (2)
C9—C14	1.379 (4)	S1—O1	1.415 (2)
C9—S1	1.739 (3)	S1—N1	1.678 (2)
C10—C11	1.373 (4)	S2—O3	1.414 (2)
C10—H10	0.9300	S2—O4	1.421 (2)
C11—C12	1.359 (5)	S2—N1	1.681 (2)
			
C2—C1—C6	122.8 (3)	C11—C12—C13	123.8 (3)
C2—C1—N1	120.6 (3)	C12—C13—C14	118.3 (3)
C6—C1—N1	116.6 (2)	C12—C13—H13	120.8
C1—C2—C3	117.6 (3)	C14—C13—H13	120.8
C1—C2—C7	121.8 (3)	C13—C14—C9	119.1 (3)
C3—C2—C7	120.6 (3)	C13—C14—H14	120.4
C4—C3—C2	119.6 (3)	C9—C14—H14	120.4
C4—C3—C8	119.5 (3)	C20—C15—C16	121.1 (3)
C2—C3—C8	120.8 (3)	C20—C15—S2	118.3 (2)
C3—C4—C5	121.5 (3)	C16—C15—S2	120.5 (2)
C3—C4—H4	119.3	C17—C16—C15	119.0 (3)
C5—C4—H4	119.3	C17—C16—H16	120.5
C6—C5—C4	120.6 (3)	C15—C16—H16	120.5
C6—C5—H5	119.7	C18—C17—C16	118.5 (3)
C4—C5—H5	119.7	C18—C17—H17	120.7
C5—C6—C1	117.8 (3)	C16—C17—H17	120.7
C5—C6—H6	121.1	F2—C18—C17	118.6 (3)
C1—C6—H6	121.1	F2—C18—C19	117.9 (3)
C2—C7—H7A	109.5	C17—C18—C19	123.5 (3)
C2—C7—H7B	109.5	C18—C19—C20	118.0 (3)
H7A—C7—H7B	109.5	C18—C19—H19	121.0
C2—C7—H7C	109.5	C20—C19—H19	121.0
H7A—C7—H7C	109.5	C19—C20—C15	119.7 (3)
H7B—C7—H7C	109.5	C19—C20—H20	120.1
C3—C8—H8A	109.5	C15—C20—H20	120.1
C3—C8—H8B	109.5	O2—S1—O1	120.26 (14)
H8A—C8—H8B	109.5	O2—S1—N1	106.76 (12)
C3—C8—H8C	109.5	O1—S1—N1	106.45 (12)
H8A—C8—H8C	109.5	O2—S1—C9	110.01 (13)
H8B—C8—H8C	109.5	O1—S1—C9	107.96 (13)
C10—C9—C14	121.2 (3)	N1—S1—C9	104.21 (11)
C10—C9—S1	119.1 (2)	O3—S2—O4	121.08 (13)
C14—C9—S1	119.7 (2)	O3—S2—N1	108.28 (13)
C11—C10—C9	119.6 (3)	O4—S2—N1	103.57 (11)
C11—C10—H10	120.2	O3—S2—C15	109.50 (13)
C9—C10—H10	120.2	O4—S2—C15	108.24 (13)
C12—C11—C10	117.9 (3)	N1—S2—C15	104.92 (11)
C12—C11—H11	121.0	C1—N1—S1	115.94 (17)
C10—C11—H11	121.0	C1—N1—S2	120.65 (17)
F1—C12—C11	118.1 (4)	S1—N1—S2	123.10 (13)
F1—C12—C13	118.1 (4)		
			
C6—C1—C2—C3	−3.5 (4)	C18—C19—C20—C15	−0.7 (4)
N1—C1—C2—C3	173.5 (2)	C16—C15—C20—C19	−1.6 (4)
C6—C1—C2—C7	176.0 (3)	S2—C15—C20—C19	176.6 (2)
N1—C1—C2—C7	−6.9 (4)	C10—C9—S1—O2	158.3 (2)
C1—C2—C3—C4	2.3 (4)	C14—C9—S1—O2	−22.4 (3)
C7—C2—C3—C4	−177.3 (3)	C10—C9—S1—O1	25.3 (3)
C1—C2—C3—C8	−177.9 (3)	C14—C9—S1—O1	−155.4 (2)
C7—C2—C3—C8	2.5 (5)	C10—C9—S1—N1	−87.6 (2)
C2—C3—C4—C5	−0.2 (5)	C14—C9—S1—N1	91.7 (2)
C8—C3—C4—C5	−179.9 (3)	C20—C15—S2—O3	158.7 (2)
C3—C4—C5—C6	−0.9 (5)	C16—C15—S2—O3	−23.2 (3)
C4—C5—C6—C1	−0.2 (5)	C20—C15—S2—O4	24.8 (2)
C2—C1—C6—C5	2.5 (5)	C16—C15—S2—O4	−157.1 (2)
N1—C1—C6—C5	−174.6 (3)	C20—C15—S2—N1	−85.3 (2)
C14—C9—C10—C11	−0.7 (4)	C16—C15—S2—N1	92.8 (2)
S1—C9—C10—C11	178.6 (2)	C2—C1—N1—S1	−98.9 (3)
C9—C10—C11—C12	−0.8 (5)	C6—C1—N1—S1	78.3 (3)
C10—C11—C12—F1	−178.5 (3)	C2—C1—N1—S2	87.2 (3)
C10—C11—C12—C13	2.4 (6)	C6—C1—N1—S2	−95.6 (3)
F1—C12—C13—C14	178.4 (3)	O2—S1—N1—C1	−166.84 (19)
C11—C12—C13—C14	−2.5 (6)	O1—S1—N1—C1	−37.2 (2)
C12—C13—C14—C9	0.9 (5)	C9—S1—N1—C1	76.7 (2)
C10—C9—C14—C13	0.6 (4)	O2—S1—N1—S2	6.85 (19)
S1—C9—C14—C13	−178.6 (2)	O1—S1—N1—S2	136.46 (16)
C20—C15—C16—C17	2.2 (4)	C9—S1—N1—S2	−109.56 (16)
S2—C15—C16—C17	−175.9 (2)	O3—S2—N1—C1	−141.3 (2)
C15—C16—C17—C18	−0.5 (4)	O4—S2—N1—C1	−11.6 (2)
C16—C17—C18—F2	178.0 (2)	C15—S2—N1—C1	101.8 (2)
C16—C17—C18—C19	−1.8 (4)	O3—S2—N1—S1	45.28 (19)
F2—C18—C19—C20	−177.4 (2)	O4—S2—N1—S1	175.00 (15)
C17—C18—C19—C20	2.4 (4)	C15—S2—N1—S1	−71.57 (18)

### Hydrogen-bond geometry (Å, º)

**Table:**

*D*—H···*A*	*D*—H	H···*A*	*D*···*A*	*D*—H···*A*
C8—H8*A*···O2^i^	0.96	2.57	3.517 (5)	170
C19—H19···O1^ii^	0.93	2.39	3.260 (4)	157

Symmetry codes: (i) *x*+1, *y*, *z*; (ii) −*x*+1, *y*−1/2, −*z*+1/2.
